# Trace metals concentrations in fresh milk from dairy farms and stores: An assessment of human health risk.

**DOI:** 10.1016/j.toxrep.2024.03.007

**Published:** 2024-03-20

**Authors:** JO Olowoyo, ML Mutemula, OO Agboola, LL Mugivhisa, OO Olatunji, OM Oladeji

**Affiliations:** a Department of Health Science and The Water School, Florida Gulf Coast University, Fort Myers, USA; b Department of Biology and Environmental Sciences, Sefako Makgatho Health Sciences University, Pretoria, South Africa; c Department of Biological Sciences, Federal University of Health Sciences, Otukpo, Benue State, Nigeria; d Department of Chemistry, University of KwaZulu Natal, South Africa

**Keywords:** Milk, Trace Metals, Estimated Health Risks, Hazard Quotient

## Abstract

Milk may be consumed daily for the supply of essential nutrients in the body, however, depending on the source, milk may contain different concentrations of trace metals. The present study investigated the presence of trace metals in fresh milk purchased from different dairy farms and stores to determine the possible health risks to humans. A total of 15 fresh milk samples were purchased from various dairy farms (7 fresh milk samples) and stores (8 milk samples). Trace metals in collected milk samples were determined using Inductively Coupled Plasma Mass Spectrometer (ICP-MS). The human health risk was determined through the Hazard Quotient (HQ), Carcinogenic and Non-carcinogenic Risk. The results showed the presence of trace metals in fresh milk stores in the following order Pb>As>Se>Cr>Ni. The highest concentration for all the elements was recorded in Mg from milk purchased from the stores and ranged from 3.37±0.16 mg/L to 4.70±0.43 mg/L. In all the milk samples analyzed, levels of As, Pb, Se, Cr, and other elements were within the acceptable range recommended by the World Health Organization (WHO). Differences obtained in the concentrations of trace metals from both the purchased milk samples and those from the dairy farms were not significant (p<0.05). The Estimated Daily Intake (EDI) and Hazard Quotient Index showed no potential health risk for each trace metal indicating no health risk for the milk consumers at this stage. The traces of trace metals in the milk samples suggest the need for regular monitoring of trace metals in milk samples because prolonged exposure to these trace metals may seriously endanger the health of consumers.

## 1 Introduction

Due to the fact that milk and dairy products in general contain all the macronutrients, which include fat, carbohydrates, vitamins, and proteins, the consumption of milk and other dairy products is on the rise[1]. Milk is regarded as a complete food [2], a nutrient-rich liquid diet which is generated by the mammary glands of mammals. Therefore, milk and its products are commonly found in our food list because of their high nutritional content, availability of vitamins and a variety of mineral elements necessary for the healthy growth and operation of different tissues and organs. [1]. In addition, fat, water, minerals, carbohydrates, proteins, vitamins, and a small amount of biological proteins and enzymes are all present in milk which also is a source of energy [3], [4]. Despite the vital advantages of milk consumption, milk may become contaminated from agricultural practices, animal feeds, the use of sewage sludge in agriculture and increasing industrial pollutants in the environment [5]. Furthermore, milk contamination also depends on the route of exposures, prevailing environmental condition, seasons, lactation stage and the breed of the animals [6].

Contamination of milk is a major threat to consumer health and needs to be addressed without delay. Mycotoxins, heavy metals, dioxins, animal feeds, and other pollutants can be hazardous to both humans and animals, making them a significant public health concern in terms of milk contamination [7], [8], [9]. Elevated pollutants in the environment have geared up the problems of milk contamination both locally and globally [10]. Globally, the contamination arising from a wide range of pollutants in the environment through different sources has proven to have a greater influence on public health and has continuously been a concern [10]. This is largely due to the symptoms, diseases and possible deaths arising from such contamination with pollutants such as heavy metals in the environment. For instance, over-exposure to heavy metals can lead to a range of ailments such as liver cirrhosis, vomiting, decrease in intelligence quotient, neurotoxicity, behavioral disorders, cancer etc. [11]. Heavy metals have been subjected to additional scrutiny due to their ability to accumulate in mammalian tissue, with previous studies revealing their presence in significant quantities in human milk and other food sources [12]. Heavy metals persist as contaminants in the environment and constitute as one of the most harmful products that can harm human health[13]. Cadmium (Cd), Arsenic (As), and Lead (Pb) are the most dangerous of these elements, resulting in human poisonings[14].

**Fig. 1:**
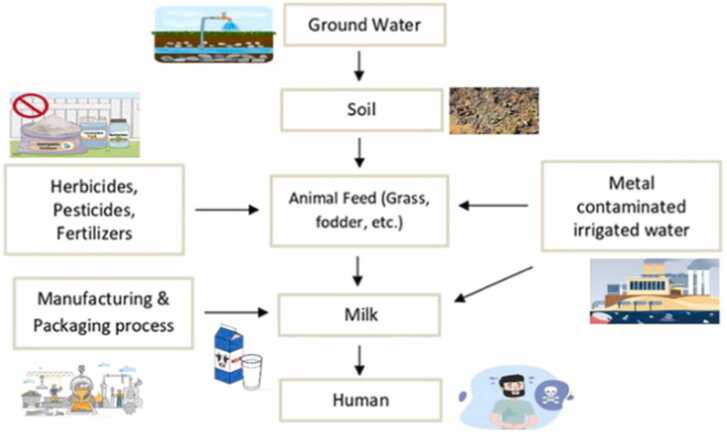
Possible food chain pathways through which humans may be exposed to trace metals through milk consumption (Modified after Islam et al. [23]).

Heavy metals can enter animal's tissues through the food chain if animal grazes on pastures irrigated with contaminated industrial waste water or drink water from a contaminated source [15]. This is because heavy metals in polluted soils can be absorbed by plants and grasses, eventually making their way into the animal's body. The study of Patra et al. [16] showed that concentrations of toxic pollutants in forages and soil significantly correlates with levels of Pb and Cd in milk of lactating cows. Purchasing fresh milk samples from animals grazed in areas with high levels of pollutants may therefore increase the risk of trace metals bioaccumulation in humans. [13], [17] (Fig. 1). Elevated concentrations of Pb (4.40 ± 1.60 mg/L) was reported by Malhat et al. [18] from raw cow milk, similarly, high level of Pb (3.80 ± 0.42 mg/L) reported in raw cow milk samples collected in Slovak. These values were all clearly above the WHO recommended limit for human consumption [19]. Heavy metals have been found in milk and other milk products in several studies, Afara et al. [17] reported high Pb concentrations in all their milk samples that exceeded the maximum permissible limit (0.02 mg/kg) established by Codex standard. Singh et al. [18] also reported high concentration of Cd and Hg in milk samples. It is quite challenging to find trace levels of metals in milk because of the intricate emulsion-like matrices, low metal ion concentrations, and complicated matrices. Farmers are obliged to ensure the safety of milk and dairy products.

Only a small number of the trace metals present in the human body, including manganese (Mn), iron (Fe), and selenium (Se), are considered to be necessary[20]. Any one of these required trace metal must be present for the body to function properly, otherwise, the body's cells will acquire particular biochemical lesions that will manifest as distinct clinical symptoms [21]. Even while certain trace metals are important for health, excessive exposure may be harmful[14], at higher concentrations to human health [22].

Previous studies have highlighted the presence of high levels of trace metals in soil and plants from some of the areas where the milk samples were purchased [24], [25]. It should also be noted that most of these farm animals do exhibit geophagia behavior which may increase the concentrations of these trace metals especially if soils high in trace metals are consumed. To the best of our knowledge, very little is known about the levels of heavy metals and trace elements in milk products from South Africa. Furthermore, additional insight into heavy metal uptake and assessment of human risks associated with milk and dairy product consumption are still required. As a result, this study was carried out to quantify the concentrations of trace elements in milk purchased from retail stores and farms, with a view to estimating health risk index associated with the consumption of trace elements through milk.

## 2 Materials and Methods

### 2.1 Chemicals

The chemicals and reagents used in this study were purchased from Merck Pty Ltd. in Johannesburg, South Africa. These included a 1000 ppm stock solution of Pb, Cd, Cu, Mg, Mn, Cr, Fe, and As. To create the diluted standard solutions, the appropriate dilution of the stock solutions was used, and all solutions were prepared with triple-distilled deionized water. Additionally, nitric acid and hydrogen peroxide for acidic digestion of samples and for cleaning containers were also supplied.

### 2.2 Sample collection and preparation

A total of 15 fresh milk samples were purchased from various dairy farms (7 milk samples) and stores (8 milk samples). The purchased samples were taken to the laboratory, without delay and samples were further divided into two groups depending on the source (farms or stores) and kept at - 20°C until the time for analysis**.** To prevent contamination, all glass tubes and bottles were cleaned, rinsed in distilled water after being soaked in 30% nitric acid, air dried, and kept clean. All milk samples were carefully washed with double-distilled water after being steeped in 1:1 nitric acid for a day.

### 2.3 Digestion of samples and estimation of heavy metals concentration

Milk samples of 25 mL were acid digested in a semi-closed glass digestion apparatus with 7 mL of HNO_3_ (Merck, South Africa) and 7 mL of 30% hydrogen peroxide (H_2_O_2_) (Merck). The resulting volume was increased to 50 mL after cooling by using distilled water. Each sample's clear filtrate was kept in the refrigerator to avoid evaporation. A blank (without sample) was prepared in the same way. Inductivity Coupled Plasma Mass Spectrometry was used to detect Mn As, Mg Cd, Se, Cr, Fe, and Pb in digested samples. Three replicates were performed for greater precision. The ICP - MS operating conditions are shown in Table 1.

**Table 1:** ICP_MS operating condition.

**Parameters**	**Settings**
Nebulizer	Standard Concentric
Nebulizer Argon Flow	0.6 L/Min
Coolant Gas Flow	12 L/min
Pump Speed	45 rpm
Purge Gas	Argon
Pump Tumbling	Tygon Orang/White
Auxiliary Gas Flow	0.5 L/Min
RF Forward Power	1150w
Center Tube	1.5 mm
Spray Chamber	Standard Cyclonic

### 2.4 Statistical analysis

The statistical analysis was performed using the Statistical Package for Social Sciences (SPSS 28.0) for windows. The differences in trace metals obtained from the two groups were determined for significance using the t – test while one way analysis of variance (ANOVA) was used within the group to separate the means at significance level of 0.05.

### 2.5 Human health risk assessment

The daily intake of metals is determined by both the content of metals in food and the amount of food consumed. Furthermore, human body weight has been shown to influence contaminant tolerance [26]. The following equation was used to calculate the estimated daily intake (EDI) of the selected heavy metals in analyzed milk [27].

(1) $$EDI=\frac{C*w}{BW}$$

Where:

C (mg/kg) denotes concentration of heavy metal in the milk,

W denotes the average daily intake (mg/person/day) and

BW is average body weight of an adult in South Africa.

### 2.6 Target hazard quotient (THQ)

Target hazard quotient is a value used to calculate the potential health risks linked to chronic exposure to heavy metals. It was determined via the following formula [28].

(2) $$THQ=\frac{EDI}{RfD}*10^{3}$$

Where RfD = oral reference dose [29] and the database which was 0.04 (Cu), 0.300 (Zn), 0.03 (Co), 0.003 (Cr), 0.0003 (As), 0.020 (Ni), 0.001 (Cd), 0.0035 (Pb), 0.70 (Fe), and 0.300 for Mn.

## 3 Results and discussion

Trace metals such as arsenic (As), selenium (Se), copper (Cu), iron (Fe), magnesium (Mg), manganese (Mn), zinc (Zn), chromium (Cr), lead (Pb), and nickel (Ni) in 15 milk samples analyzed in this study are shown in Table 2a for milk from the farms and Table 2b for milk from the retail stores. The concentrations of trace elements in the farms and stores were found to be in the order Mg>Fe>Zn>Cu. Meanwhile, the concentrations of heavy metals in fresh milk from stores were observed to be Pb>As>Se>Cr>Ni. Previous studies have suggested variations in the concentrations of heavy metals in milk products which is like those reported in our study and linked the presence of these heavy metals to different environmental factors. [30]

**Table 2a:** The concentration levels of heavy metals (mg/L) in fresh milk bought from dairy farms in Pretoria.

Samples	As	Cr	Cu	Fe	Mg	Mn	Ni	Pb	Se	Zn
A	0.029±0.017	0.024±0.017	0.010±0.004	0.268±0.032	3.355±0.363	0.008±0.001	0.003±0.005	0.072±0.079	0.035±0.041	0.185±0.012
B	0.029±0.017	0.008±0.007	0.012±0.004	0.282±0.038	3.631±0.379	0.007±0.001	0.004±0.005	0.018±0.002	0.021±0.023	0.293±0.034
C	0.026±0.018	0.007±0.008	0.012±0.004	0.278±0.020	3.384±0.255	0.008±0.001	0.004±0.006	0.020±0.004	0.015±0.016	0.309±0.036
D	0.025±0.015	0.005±0.008	0.011±0.004	0.239±0.022	3.597±0.221	0.007±0.001	0.003±0.005	0.016±0.003	0.012±0.013	0.180±0.008
E	0.018±0.018	0.001±0.022	0.011±0.003	0.297±0.017	3.897±0.204	0.008±0.001	0.006±0.008	0.018±0.003	0.025±0.032	0.197±0.020
F	0.021±0.010	0.004±0.010	0.009±0.003	0.346±0.046	3.731±0.207	0.007±0.001	0.004±0.006	0.021±0.004	0.013±0.015	0.200±0.052
G	0.019±0.011	0.004±0.010	0.011±0.005	0.305±0.019	3.859±0.214	0.007±0.001	0.003±0.004	0.022±0.003	0.013±0.015	0.220±0.079
Range	**0.018±0.018–0.029±0.017**	**0.001±0.022–0.024±0.017**	**0.009±0.003–0.012±0.004**	**0.239±0.022–0.346±0.046**	**3.355±0.363–3.897±0.204**	**0.007±0.001–0.008±0.001**	**0.003±0.004–0.006±0.008**	**0.016±0.003–0.072±0.079**	**0.012±0.013–0.035±0.041**	**0.185±0.012–0.309±0.036**
WHO Recommended limits	0.01	0.05	1.50	0.5	150	0.40	0.02	0.01	0.02	5.00

**Table 2b:** The concentration levels of heavy metals (mg/L) in fresh milk bought from stores in Pretoria.

Samples	As	Cr	Cu	Fe	Mg	Mn	Ni	Pb	Se	Zn
A	0.025±0.017	0.007±0.010	0.013±0.006	0.242±0.067	3.922±0.308	0.009±0.001	0.006±0.007	0.020±0.005	0.047±0.052	0.218±0.053
B	0.021±0.033	0.019±0.011	0.015±0.009	0.284±0.093	3.539±0.248	0.007±0.001	0.009±0.007	0.014±0.007	0.060±0.006	0.229±0.026
C	0.029±0.041	0.021±0.014	0.018±0.010	0.430±0.142	3.373±0.161	0.016±0.001	0.009±0.007	0.013±0.008	0.048±0.006	0.262±0.040
D	0.027±0.037	0.022±0.015	0.023±0.012	0.297±0.015	3.938±0.237	0.015±0.001	0.008±0.006	0.036±0.018	0.049±0.005	0.297±0.076
E	0.027±0.038	0.026±0.016	0.018±0.015	0.274±0.049	4.066±0.253	0.010±0.003	0.017±0.016	0.014±0.009	0.041±0.008	0.364±0.167
F	0.025±0.036	0.025±0.016	0.011±0.006	0.288±0.022	3.535±0.217	0.010±0.001	0.008±0.006	0.013±0.007	0.040±0.008	0.199±0.006
G	0.029±0.039	0.022±0.016	0.025±0.006	0.434±0.013	4.704±0.432	0.019±0.001	0.006±0.005	0.021±0.003	0.049±0.005	0.247±0.038
H	0.028±0.038	0.026±0.017	0.019±0.012	0.427±0.143	4.145±0.448	0.018±0.009	0.006±0.005	0.013±0.009	0.047±0.006	0.215±0.020
**Range**	**0.021±0.033** **-0.029±0.041**	**0.007±0.010** **-0.026±0.016**	**0.011±0.006** **-0.025±0.006**	**0.242±0.067** **-0.434±0.013**	**3.373±0.161** **-4.704±0.432**	**0.007±0.001** **-0.019±0.001**	**0.006±0.005** **-0.017±0.016**	**0.013±0.007** **-0.036±0.018**	**0.040±0.008** **-0.060±0.006**	**0.199±0.006** **-0.364±0.167**
WHO Recommended limits	0.01	0.05	1.50	0.5	150	0.40	0.02	0.01	0.02	5.00

The concentration of Mg in both the farm milk and milk from stores ranged from 3.35±0.36 mg/L - 4.70±0.43 mg/L. Mg in particular plays a crucial role in human body processes [31], and is the fourth-most prevalent cation in the body[32]. It has been demonstrated that Mg can assist in controlling inflammation, cell proliferation, and glucose metabolism [33]. Mg and Ca compete with one another when consumed for intestine intraepithelial absorption and renal reabsorption[31]. Low quantities of either Mg or Ca could intensify the effects of the other since their body concentrations are controlled by a negative feedback mechanism [33]. In the present study, all the values recorded for Mg were below the WHO permissible limit of 150 mg/L [34]. The estimated daily intake for Mg ranged from (6.99–9.80 mg/day) (Table 3a) for the samples from the farm and from the stores. The HQ was below 1, which means that it is unlikely to cause any adverse health effects.

The concentration of Fe in the analyzed milk samples ranged from 0.239±0.022 mg/L to 0.346±0.046 mg/L in dairy farm milk and 0.242±0.067 mg/L- 0.434±0.013 mg/L in the milk purchased from stores. The values of Fe in all the milk samples in the current study were lower than the values reported by Simsek et al. [35]which ranged from 0.80 mg/kg to 5.30 mg/kg but higher than some of those reported by Pilarezyk et al. [36] which ranged from 0.140 to 0.497 µg/mL. The concentrations of the heavy metals in all milk samples were lower than the WHO recommended permissible limit of 0.5 mg/kg [37]. Estimated daily intake for Fe ranged from (0.50–0.90 mg/day) which is below the RDA of 8 mg/day [38]. The Hazard quotient was below 1 which means that there is no health risk from Fe at this stage.

The levels of Pb in the milk samples ranged from 0.016±0.00 mg/L-0.072±0.08 mg/L in the milk samples from the farm and 0.01±0.01 mg/L - 0.04±0.02 mg/L in the milk brought from the stores (Table 1 and 2). The highest concentration was reported from one of the raw milk samples used in this study. Lead (Pb) is classified as a trace metal that is not beneficial for human health and primarily affects human nervous and vascular systems [40]. Pb poisoning can cause anemia, lethargy, kidney and brain damage, as well as death [41]. Due to lead's ability to pass the placental barrier, pregnant women who are exposed to it expose their unborn children to it as well [42]. Excessive lead exposure has the potential to be lethal [43]. Malhat *et al*. [44] reported Pb concentrations in milk collected from different places in Egypt which ranged from 1.850 mg/L to 4.404 mg/L. These concentrations were higher than the concentrations reported in this study. The EDI for Pb ranged from 0.003 to 0.15 mg/L in the sample brought from stores and the dairy farm. The hazard quotient was below 1 which means that there is no health risk to the milk consumers at this stage but may require constant monitoring due to its presence in the milk samples used in this study.

Zinc concentrations ranged from 0.185±0.012 mg/L - 0.309±0.036 mg/L in milk from dairy farms and 0.199±0.006 mg/L- 0.364±0.167 mg/L from milk purchased from stores (Table 1 and 2). These concentrations were lower than the permissible limit set by the WHO which is 5.0 mg/L[37]. The concentrations in the current study, were lower than the concentrations reported by Simsek et al. [35] and Licata et al. [45] which are 2.30–6.46 mg/L and 0.024–4.961 mg/L respectively. The estimated daily intake recorded for all the samples was 0.04 mg/day for both samples and was lower than the RDA which is 11 mg/day for men and 8 mg/day for females [46]. The HQ was also lower than 1. Zinc is a primary structural component of cells and regulates the activity of several enzymes [47]. It also has antioxidant qualities and helps to keep the redox balance in cells in check [48]. The negative effects of high Zn levels in the body can cause adverse effects such as nausea, vomiting, loss of appetite, abdominal cramps, and diarrhea [49].

The concentrations reported for Cu ranged from 0.009±0.003 mg/L-0.012±0.004 mg/L milk from the dairy farm and 0.011±0.006 mg/L - 0.025±0.006 mg/L in milk brought from stores (Table 1 and 2). The highest concentration was recorded in milk purchased from one of the stores. Also, the concentrations of Cu are lower than the permissible limit set by the WHO which is 1.5 mg/L. Copper is a crucial trace element vital for proper growth [50]. The EDI recorded for Cu was 0.02–0.05 mg/day which is lower than the RDA limit of 900 µg/day for Cu. Based on the present results the hazard quotient is lower than 1 which means that there is no risk associated with the intake of milk used for this study.

Arsenic levels ranged from 0.018±0.018 mg/L- 0.029±0.017 mg/L for milk purchased from the farm and 0.021±0.033 mg/L - 0.029±0.04 mg/L in all the milk purchased from stores. The concentrations of the present study were just around the WHO permissible limit of 0.01 ppm [51]. Constant monitoring and elucidation of factors that are responsible for the values obtained for As in this study is important. Carrera et al. [52] also determined the concentration of As in cow milk samples collected from Cordoba and the levels of As in their samples ranged from 0.3 to 10.5 mg/L which is higher than the values of the current study. The EDI (0.04–0.06 mg/day) was less than the recommended RDA of 0.3 g/kg/day.

Chromium is essential to maintain the metabolic systems of the human body and can lead to poisoning at higher levels [53]. Cr is detected in all the samples analyzed with the concentration ranging from 0.001±0.022 mg/L- 0.024±0.017 mg/L and 0.007±0.010 mg/L-0.026±0.016 mg/L in milk from farms and stores respectively (Table 1 and 2). Chromium (Cr) levels in the present study were found to be lower than the permissible limit of 0.05 mg/L established by the WHO [37]. According to Muhib [28] the highest concentrations of Cr present in branded milk and dairy cow milk in Bangladesh were recorded at 0.672±0.010 mg/L and 0.373±0.008 mg/L respectively, which are higher than the result of the present study (0.001±0.022 mg/L- 0.024±0.017 mg/L and 0.007±0.010 mg/L-0.026±0.016 mg/L). The adequate daily dietary intake of 20–30 µg/day has been set [54], and in this study, the EDI for Cr was (0.00–0.05 mg/day), and the health hazard risk was found to be less than one, indicating that it is unlikely to cause any health risk at this stage.

The levels of Nickel (Ni) ranged from 0.003±0.004 mg/L-0.006±0.008 mg/L in milk from the farm and 0.006±0.005 mg/L-0.017±0.016 mg/L in the milk from stores (Table 1 and 2). These concentrations were below the permissible limit of WHO which is 0.02 mg/L [34]. The highest concentration of Ni was recorded in milk from stores with a concentration of 0.017±0.016 mg/L. Nickel's intestinal absorption varies significantly depending on its chemical form and can rise when there is a lack of iron[55]. Ni can be excreted in milk after overcoming the placental barrier [56]. The hazard quotient is lower than 1, therefore it is unlikely to cause non-carcinogenic health effects in people consuming the milk. The recommended dietary allowance for Ni has not yet been established [57].

Also, concentrations of Mn ranged from 0.007±0.001 mg/L – 0.019 ± 0.001 mg/L from stores and 0.007±0.001 mg/L – 0.008±0.001 mg/L in the milk purchased from the dairy farms (Table 1 and 2). These concentrations are lower than the permissible limit set by WHO of 0.40 ppm [39]. High manganese exposure may cause decreased neurological and neuromuscular control, as well as mental and emotional disorders (muscle stiffness, and lack of coordination. Extremely high doses may affect male fertility, cause birth abnormalities, and affect bone formation [58]. A neurodegenerative illness characterized by anomalies of the central nervous system and neuropsychiatric disorders has been linked to chronic and excessive Mn inhalation [59], but in the present study the concentrations were above the permissible limit. The current EDI of Mn (0.01–0.04 mg/day) in this study is below the Environmental Protection Agency (EPA) established standard of 0.3 µg/kg/day[60], and the hazard quotient is below 1. Thus, the risk is unlikely to cause any non-carcinogenic health effects.

**Fig. 2:**
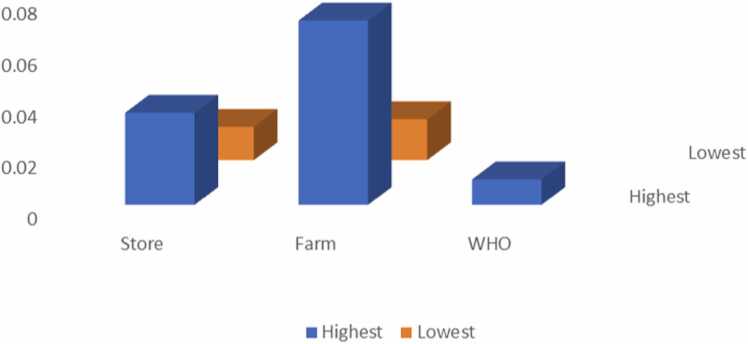
Showing the highest and lowest concentrations of Pb from the milk samples.

The concentrations of Se in the present study ranged from 0.012±0.013 mg/L-0.035±0.041 mg/L from milk from the farm and 0.040±0.008 mg/L-0.060±0.006 mg/L from stores (Table 1 and 2). The highest concentration was recorded in milk bought from the stores with a concentration of 0.060±0.006 mg/L. Taking in Se over an extended length of time may result in selenosis, a condition characterized by hair loss, fragile nails, and numbness of the limbs [61], [62]. In most of the samples, Se levels were above the World Health Organization’s permissible limit of 0.02 mg/L [63]. The estimated daily intake of Se is lower than the recommended dietary allowance which is 55 µg/day [64], and the hazard quotient was below 1, therefore, there is no chance of causing non-carcinogenic health effects. Fig 2

### 3.1 Target hazard quotient (THQ)

The presence of heavy metals in milk products was below the threshold values. The estimated daily intake (EDI) and Target Hazard Quotient (THQ) values of heavy metals via the consumption of milk were calculated and shown in Table 3a, Table 3b. THQ of 1 indicates that the exposed population is safe going by the values obtained for EDI and THQ for all the samples. In the current investigation, the THQ value for each metal was found to be below one (Table 3a, Table 3b) for all collected milk samples. The findings are in agreement with Islam et al. [65] and Mihib et al. [28]. THQ >1 indicates acute health effects in consumers[65].

**Table 3a:** Estimated Dairy Intake and Hazard Quotient in fresh milk bought from dairy farms in Pretoria.

	As EDI	HQ	Cr EDI	HQ	Cu EDI	HQ	Fe EDI	HQ	Mg EDI	HQ	Mn EDI	HQ	Ni EDI	HQ	Pb EDI	HQ	Se EDI	HQ	Zn EDI	HQ
1	0.06	0.201	0.05	0.017	0.02	0.001	0.56	0.001	6.99	0.049	0.02	5.55E-05	0.01	3.12E-4	0.15	0.042	0.07	0.014	0.04	1.16 E-4
2	0.06	0.201	0.02	0.005	0.03	0.001	0.59	0.001	7.56	0.054	0.01	4.86E-05	0.01	4.16E-4	0.04	0.011	0.04	0.008	0.04	1.16 E-4
3	0.05	0.181	0.01	0.005	0.03	0.001	0.58	0.001	7.05	0.050	0.02	5.55E-05	0.01	4.16 E-4	0.04	0.011	0.03	0.006	0.04	1.16 E-4
4	0.05	0.174	0.01	0.003	0.02	0.001	0.50	0.001	7.49	0.054	0.01	4.86E-05	0.01	3.12 E-4	0.03	0.009	0.03	0.005	0.04	1.16 E-4
5	0.04	0.125	0.00	0.001	0.02	0.001	0.62	0.001	8.12	0.058	0.02	5.55E-05	0.01	6.25 E-4	0.04	0.010	0.05	0.010	0.04	1.16 E-4
6	0.04	0.146	0.01	0.003	0.02	0.000	0.73	0.001	7.77	0.055	0.01	4.86E-05	0.01	4.16 E-4	0.04	0.0125	0.03	0.005	0.04	1.16 E-4
7	0.04	0.132	0.01	0.003	0.02	0.001	0.64	0.001	8.04	0.057	0.01	4.86E-05	0.01	3.12 E-4	0.05	0.013	0.03	0.005	0.04	1.16 E-4

**Table 3b:** Estimated Dairy Intake and Hazard quotient in milk bought from stores in Pretoria.

	As EDI	HQ	Cr EDI	HQ	Cu EDI	HQ	Fe EDI	HQ	Mg EDI	HQ	Mn EDI	HQ	Ni EDI	HQ	Pb EDI	HQ	Se EDI	HQ	Zn EDI	HQ
1	0.05	0.174	0.01	0.005	0.03	0.001	0.50	0.001	8.17	0.058	0.02	0.000	0.01	0.001	0.04	0.012	0.10	0.020	0.04	0.000
2	0.04	0.146	0.04	0.013	0.03	0.001	0.52	0.001	7.37	0.053	0.01	0.000	0.02	0.001	0.03	0.008	0.13	0.025	0.04	0.000
3	0.06	0.201	0.04	0.015	0.04	0.001	0.90	0.001	7.03	0.050	0.03	0.000	0.02	0.001	0.03	0.008	0.10	0.020	0.04	0.000
4	0.06	0.188	0.05	0.015	0.05	0.001	0.62	0.001	8.20	0.059	0.03	0.000	0.02	0.001	0.08	0.021	0.10	0.020	0.04	0.000
5	0.06	0.188	0.05	0.018	0.04	0.001	0.57	0.001	8.47	0.061	0.02	0.000	0.04	0.002	0.03	0.008	0.09	0.017	0.04	0.000
6	0.05	0.174	0.05	0.017	0.02	0.001	0.60	0.001	7.36	0.053	0.02	0.000	0.02	0.001	0.03	0.008	0.08	0.017	0.04	0.000
7	0.06	0.201	0.05	0.015	0.05	0.001	0.90	0.001	9.80	0.070	0.04	0.000	0.01	0.001	0.04	0.013	0.10	0.020	0.04	0.000
8	0.06	0.194	0.05	0.018	0.04	0.001	0.89	0.001	8.64	0.062	0.04	0.000	0.01	0.001	0.03	0.008	0.10	0.020	0.04	0.000

## 4 Conclusion

The current research was designed to determine the concentrations of trace elements from both the raw milk products and the processed milk products purchased from different stores. Trace metals and elements examined in this study were either lower or within the acceptable limit for human consumption. However, their presence in these milk products should necessitate a continuous monitoring program that will assist in tracking the values of these trace metals periodically to prevent adverse effects on human health. In some instances, the trace metals content of milk samples purchased from the dairy farms have values greater than the processed milk though the differences were not significant. Most of the trace elements analyzed in milk samples from the farms and stores were below the World Health Organization permissible limits of trace metals in milk consumed by people. The low concentrations recorded for most of the trace metals might have accounted for its current status of safety if the Target Hazard Quotient values of <1 are considered. However, since the trace metals do not get biodegraded easily, long-term exposure to these trace metals can cause serious health problems to people consuming the milk especially if there are other exposure routes which that were not examined in this study. It is important to regularly monitor the concentrations of trace metals in milk especially from dairy farms since different factors as pointed out in this study and in literature could elevate the concentrations of the trace elements in the milk.

## CRediT authorship contribution statement

**Muhulisi Lusani Mutemula:** Investigation, Methodology, Writing – original draft. **Joshua Oluwole Olowoyo:** Conceptualization, Project administration, Writing – review & editing. **Olatunji OO:** Investigation, Methodology. **LL Mugivhisa:** Writing – review & editing. **Agboola OO:** Writing – review & editing. **Oluwaseun Mary Oladeji:** Formal analysis, Project administration, Writing – review & editing.

## Declaration of Competing Interest

The authors declare that they have no known competing financial interests or personal relationships that could have appeared to influence the work reported in this paper.

## Data Availability

Data will be made available on request.
